# Supplementary material for: Content-rich biological network constructed by mining PubMed abstracts
Source: BMC Bioinformatics. 2004 Oct 8;5:147. doi: 10.1186/1471-2105-5-147 (PMC528731; doi:10.1186/1471-2105-5-147)
Supplement: Additional File 2 — The original results of the above study (non-essential files are deleted to keep the file size under the limit set by BMC bioinformatics). [file 1471-2105-5-147-S2.bz2 › chilibotAdditionalFile2/dip05/690ID10393292E18011/html/CHEY_ARCB.html]

 


 **CHEY** and **ARCB** 
  
Found 5 abstracts in PubMed, retrieved 05.  
 

 What does Google say? 
 PDF only 
| .edu only 

---

**Interactive relationship** (e.g. stimulation, inhibition, etc)

**Neutral relationship**- Interaction between the  **CheY**  response regulator and the histidine containing phosphotransfer HPt domain of the  **ArcB**  sensory kinase in Escherichia coli.  Ref: 9188789 FEBS Lett, 1997
- Structure of the histidine containing phosphotransfer HPt domain of the anaerobic sensor protein  **ArcB**  complexed with the chemotaxis response regulator  **CheY** .  Ref: 10393292 Acta Crystallogr D Biol Crystallogr, 1999
- The histidine containing phosphotransfer HPt domain at the C terminus of the anaerobic sensor kinase  **ArcB**  has been cocrystallized with the chemotaxis response regulator  **CheY**  by a hanging drop vapor diffusion method.  Ref: 9761838 Acta Crystallogr D Biol Crystallogr, 1998

**Non-interactive relationship** (e.g. studied together, co-existance, homology, etc.)

- The three dimensional structure of the HPt domain of  **ArcB**  complexed with  **CheY**  has been determined using the molecular replacement method.  Ref: 10393292 Acta Crystallogr D Biol Crystallogr, 1999
- Crystallization of a complex between a novel C terminal transmitter, HPt domain, of the anaerobic sensor kinase  **ArcB**  and the chemotaxis response regulator  **CheY** .  Ref: 9761838 Acta Crystallogr D Biol Crystallogr, 1998
